# Antioxidant and Antiproliferative Activities of the Essential Oils from *Thymbra capitata* and *Thymus* Species Grown in Portugal

**DOI:** 10.1155/2015/851721

**Published:** 2015-07-02

**Authors:** Maria Graça Miguel, Custódia Gago, Maria Dulce Antunes, Cristina Megías, Isabel Cortés-Giraldo, Javier Vioque, A. Sofia Lima, A. Cristina Figueiredo

**Affiliations:** ^1^Departamento de Química e Farmácia, Faculdade de Ciências e Tecnologia, Universidade do Algarve, MeditBio, Campus de Gambelas, 8005-139 Faro, Portugal; ^2^Instituto de la Grasa (C.S.I.C.), Universidad Pablo de Olavide, Edificio 46, Carretera de Utrera, km 1, 41013 Sevilla, Spain; ^3^Centro de Estudos do Ambiente e do Mar Lisboa, Faculdade de Ciências, Universidade de Lisboa, CBV, DBV, 1749-016 Lisboa, Portugal; ^4^Instituto Politécnico de Bragança, Escola Superior Agrária, Centro de Investigação de Montanha, Campus de Santa Apolónia, Apartado 1172, 5301-855 Bragança, Portugal

## Abstract

The antioxidant and antiproliferative activities of the essential oils from *Thymbra capitata* and *Thymus* species grown in Portugal were evaluated. *Thymbra* and *Thymus* essential oils were grouped into two clusters: Cluster I in which carvacrol, thymol, *p*-cymene, *α*-terpineol, and *γ*-terpinene dominated and Cluster II in which thymol and carvacrol were absent and the main constituent was linalool. The ability for scavenging ABTS^•+^ and peroxyl free radicals as well as for preventing the growth of THP-1 leukemia cells was better in essential oils with the highest contents of thymol and carvacrol. These results show the importance of these two terpene-phenolic compounds as antioxidants and cytotoxic agents against THP-1 cells.

## 1. Introduction

*Thymbra capitata* and several* Thymus* species grown in Portugal produce essential oils (EOs) of interest for the food and fragrance industries and are also of medicinal value. Opposite to essential oils of* T. capitata*, characterized by a great chemical homogeneity with high carvacrol relative amounts,* Thymus* EOs show many chemotypes [1].

Although this chemical polymorphism may represent a problem for the required efficacy constancy of an EO, the EOs isolated from* T. capitata* and from different Portuguese* Thymus* species have all been shown to possess anti-inflammatory, antimicrobial, antioxidant, antiparasitical, insecticidal, and nematicidal activity, among other biological properties [1–10].

Earlier studies have shown the antioxidant potential of these EOs, but no previous report addressed the antiproliferative properties of the EOs from* T. capitata* and* Thymus* species grown in Portugal. For this reason, the main goal of the present work was to determine the antiproliferative activity of these EOs on the THP-1 leukemia cell line. Also, the* in vitro* antioxidant activity was evaluated with methodologies based on distinct mechanisms: one based on electron transfer and the other on hydrogen atom transfer (Trolox Equivalent Antioxidant Capacity (TEAC) and Oxygen Radical Antioxidant Capacity (ORAC), resp.).

## 2. Material and Methods

### 2.1. Plant Material

The aerial parts of Portuguese* Thymbra* and* Thymus* species, from collective or individual samples, were collected from wild-grown plants in the mainland of Portugal and in the Azores archipelago (Portugal). Plant material was stored at −20°C until extraction. In total, EOs isolated from 9 plant samples were evaluated for chemical composition and biological activity (Table 1). Certified voucher specimens have been deposited at the Herbarium of the Botanical Garden of Lisbon University (Lisbon, Portugal).

### 2.2. Isolation and Chemical Analysis of the EOs

Essential oils were isolated from fresh plant material by hydrodistillation for 3 h, using a Clevenger-type apparatus, according to the European Pharmacopoeia [11], and analyzed by gas chromatography (GC), for component quantification, and gas chromatography coupled to mass spectrometry (GC-MS) for component identification, as detailed in Barbosa et al. [2]. Gas chromatographic analyses were performed using a Perkin Elmer Autosystem XL gas chromatograph (Perkin Elmer, Shelton, CT, USA) equipped with two flame ionization detectors (FIDs), a data handling system, and a vaporizing injector port into which two columns of different polarities were installed: a DB-1 fused-silica column (30 m × 0.25 mm i.d., film thickness 0.25 *μ*m; J&W Scientific Inc., Rancho Cordova, CA, USA) and a DB-17HT fused-silica column (30 m × 0.25 mm i.d., film thickness 0.15 *μ*m; J&W Scientific Inc.). Oven temperature was programmed to increase from 45 to 175°C, in 3°C/min increments, and then up to 300°C in 15°C/min increments and finally held isothermal for 10 min. Gas chromatographic settings were as follows: injector and detectors temperatures, 280°C and 300°C, respectively; carrier gas, hydrogen, adjusted to a linear velocity of 30 cm/s. The samples were injected using a split sampling technique, ratio 1 : 50. The volume of injection was 0.1 *μ*L of a pentane-oil solution (1 : 1). The percentage composition of the oils was computed by the normalization method from the GC peak areas, calculated as a mean value of two injections from each oil, without response factors. The GC-MS unit consisted of a Perkin Elmer Autosystem XL gas chromatograph, equipped with DB-1 fused-silica column (30 m × 0.25 mm i.d., film thickness 0.25 *μ*m; J&W Scientific, Inc.) interfaced with Perkin-Elmer Turbomass mass spectrometer (software version 4.1, Perkin Elmer). GC-MS settings were as follows: injector and oven temperatures, as above; transfer line temperature, 280°C; ion source temperature, 220°C; carrier gas, helium, adjusted to a linear velocity of 30 cm/s; split ratio, 1 : 40; ionization energy, 70 eV; scan range, 40–300 u; scan time, 1 s. The identity of the components was assigned by comparison of their retention indices relative to C_9_–C_21_ *n*-alkane indices, and GC-MS spectra from a laboratory made library based upon the analyses of reference oils, laboratory-synthesized components, and commercial available standards. The percentage composition of the isolated EOs was used to determine the relationship between the different samples by cluster analysis using NTSYS, and the degree of correlation was graded as very high (0.9-1), high (0.7–0.89), moderate (0.4–0.69), low (0.2–0.39), and very low (<0.2), as detailed in Faria et al. [12].

### 2.3. Antioxidant Activity

#### 2.3.1. ABTS^•+^ Free Radical Scavenging Activity

The determination of ABTS^•+^ radical scavenging was carried out as described in Antunes et al. [13]. The absorbance was monitored spectrophotometrically at 735 nm for 6 min with a Shimadzu spectrophotometer 160-UV. The antioxidant activity of each sample was calculated as scavenging effect % (IA%) = (1 − *A*_*f*_/*A*_0_) × 100, where *A*_0_ is absorbance of the control and *A*_*f*_ the absorbance in the presence of the sample. The values were compared with the curve for several Trolox concentrations and the values given as mM Trolox Equivalent Antioxidant Capacity.

#### 2.3.2. Oxygen Radical Absorbance Capacity (ORAC) for EOs

Fluorescein (FL) was the fluorescent probe used in the ORAC method, as described by Ou et al. [14]. EOs samples were diluted 1000 times in acetone before analysis. The equipment used was a Tecan Infinite M200 Microplate Reader. ORAC values were calculated according to [15]. Briefly, the net area under the curve (AUC) of the standards and samples was calculated. The standard curve was obtained by plotting Trolox concentrations against the average net AUC of the three measurements for each concentration. Final ORAC values were calculated using the regression equation between Trolox concentration and the net AUC and were expressed as *μ*mol Trolox/g EO. Tests were carried out in triplicate.

### 2.4. Antiproliferative Activity

#### 2.4.1. Cell Culture

THP-1 cells were cultured in Dulbecco's Modified Eagle Medium (DMEM) supplemented with 10% (v/v) foetal bovine serum, 1% (v/v) nonessential amino acids, 100 U/mL penicillin, and 100 *μ*g/mL streptomycin. Cells were incubated at 37°C in a humidified 5% CO_2_ atmosphere.

#### 2.4.2. Antiproliferative Activity Evaluation

The growth-inhibitory effect of EOs was measured using the 3-(4,5-dimethylthiazol-2-yl) 2,5-diphenyltetrazolium bromide (MTT) assay adopted from Mosmann [16]. THP-1 cells were seeded in 96-well plate at 5 · 10^3^ cells/well and exposed to different concentrations of EOs (10–500 *μ*g/mL) for 1 and 4 days. All test substances were dissolved in dimethyl-sulphoxide (DMSO). The solvent concentration in the incubation medium never exceeded 0.5%. Control cultures received the equivalent concentration of DMSO. After treatment, cells were incubated for 1 h in the usual culture conditions after addition of the same volume of medium containing MTT (2 mg/mL). After this incubation, 150 *μ*L HCl (0.1 M) in isopropanol was added to dissolve the blue formazan crystals formed by reduction of MTT. Absorbance at 570 nm using a background reference wavelength of 630 nm was measured using a dual-wavelength Multiskan Spectrum (Thermo) plate reader. The mean absorbance values for the negative control (DMSO treated cells) were standardized as 100% absorbance (i.e., no growth inhibition) and results were displayed as absorbance (% of control)* versus* essential oil concentration. Tests were carried out in triplicate.

### 2.5. Statistical Analysis

Data were analysed by one-way analysis of variance (ANOVA) using IBM SPSS Statistics version 20. Tukey's test was used to determine the difference at 5% significance level. Paired Student's *t*-test was used in some tests to determine differences at 5% significance.

## 3. Results and Discussion

### 3.1. Chemical Composition of the EOs

The identified components in the 9 EOs isolated from* Thymbra* and* Thymus* species, from mainland Portugal and Azores islands, are listed in Table 2 in their elution order on the DB-1 GC column, arranged according to the degree of correlation obtained after agglomerative cluster analysis based on the EOs chemical composition.

**Figure 1:**
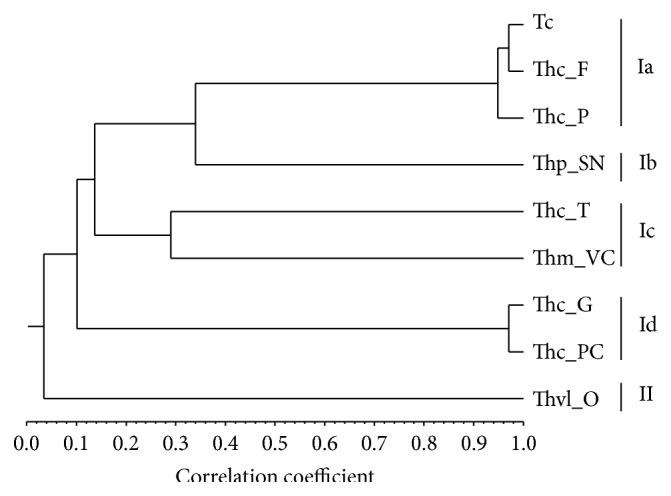
Dendrogram obtained by cluster analysis of the percentage composition of the essential oils isolated from* Thymbra capitata* and* Thymus* species based on correlation and using unweighted pair-group method with arithmetic average (UPGMA). For abbreviations, see Table 1.

Two poorly correlated clusters (*S*_corr_ < 0.2) could be identified, Clusters I and II. Cluster I was subdivided into four subclusters (Figure 1 and Table 2). Cluster I grouped eight of the nine samples, all having in common variable percentages of carvacrol (0.1–71%), *α*-terpineol (0.1–44%), thymol (traces-42%),* p*-cymene (6–14%), and *γ*-terpinene (3–11%). Cluster Ia was characterized by dominance of carvacrol (46–71%), whereas in Cluster Ib predominated geraniol (33%), not present in most of the remaining samples. Thymol (14–42%) was the main component in Cluster Ic and *α*-terpineol (36–44%) in Cluster Id. Thymol and carvacrol were not detected in Cluster II, which was dominated by linalool (66%).

With variable amounts, these results are in accordance with previous studies on* T. capitata* as well as* Thymus* species grown in Portugal (for references, see Section 1).

### 3.2. Antioxidant Activity

The EOs antioxidant activity was assessed using two methods, based on two distinct mechanisms: electron reaction-based method (TEAC) and hydrogen reaction-based method (ORAC).

Using TEAC method, the EO isolated from* Th. caespititius* collected in Terceira (Thc_T) showed the highest antioxidant activity (27.3 *μ*mol TE/g EO) in contrast to the lowest antioxidant activity of* Th. villosus* EO (Thvl_O: 3.7 *μ*mol TE/g EO). Large activity differences were observed among the 5* Th. caespititius* EOs assessed (Table 2), with those isolated from plant material collected in mainland Portugal (Praia do Cortiço and Gerês) showing the lowest ability for scavenging ABTS radicals.

The lowest activities observed in* Th. villosus* and the two* Th. caespititius* EOs may be related with their main components: linalool and *α*-terpineol, respectively (Tables 2 and 3), whereas the EOs with highest activity were dominated by thymol (Thc_T) and carvacrol (Tc, Thc_F, and Thc_P). Although geraniol and 1,8-cineole predominated in* Th. pulegioides* (Thp_SN) and* Th. mastichina* (Thm_VC) EOs, they showed also relatively high percentages of thymol and carvacrol which may contribute to their scavenging capacity of ABTS (Table 2).

Dandlen et al. [3] did not observe correlation between* Th. caespititius* main EO component and the antioxidant activity after assaying the antioxidant activities of six Portuguese thyme EOs, by four methods: thiobarbituric acid reactive substances (TBARS), free radical scavenging activity through the capacity for scavenging DPPH (2,2-diphenyl-1-picryl-hydrazyl), and the hydroxyl and superoxide anion radicals' scavenging. Indeed, in some cases, the same main component in different EOs of the same species but collected in different places of Portugal had different abilities for scavenging the free radicals and/or preventing lipid peroxidation. In the present work, in the group of* Th. caespititius* EOs, the highest activities were always in those in which thymol (Terceira) or carvacrol (Faial, Pico) prevailed (Tables 1 and 2).

As it was observed with the TEAC method, all thymol and carvacrol rich EOs (Thc_T, Thc_F, and Tc, Tables 2 and 3) showed also the highest scavenging peroxyl radicals capacity, by the ORAC method. Linalool and *α*-terpineol rich EOs (Thc_PC, Thc_G, and Thvl_O, Tables 2 and 3) showed the lowest activity.

Thymol and carvacrol's higher capacity for scavenging peroxyl radicals than linalool and 1,8-cineole was previously reported [17, 18]. In contrast to the results obtained in the present work, *α*-terpineol was considered by Bicas et al. [19] as possessing good capacity for scavenging peroxyl radicals. Since EOs are a complex mixture, this may reflect the presence of some other components that interfere with the capacity of this oxygenated monoterpene for scavenging peroxyl radicals.

### 3.3. Antiproliferative Activity

**Figure 2:**
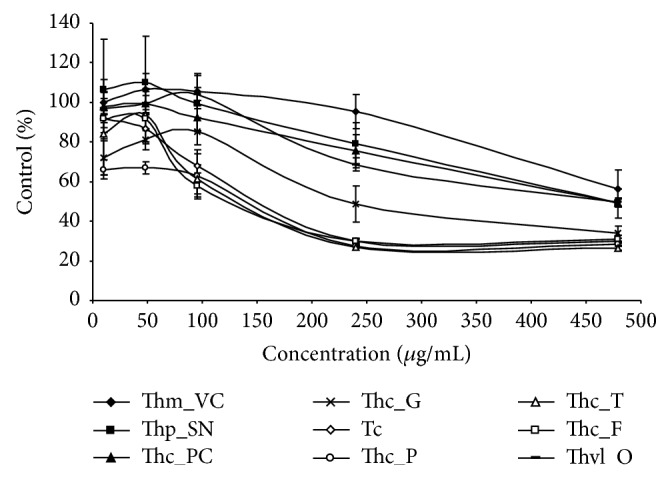
Antiproliferative activity of the essential oils on THP-1 cell line with 24 h exposure. The mean absorbance values for the negative control (DMSO treated cells) were standardized as 100% absorbance (i.e., no growth inhibition) and results were displayed as absorbance (% of control)* versus* essential oil concentration.

**Figure 3:**
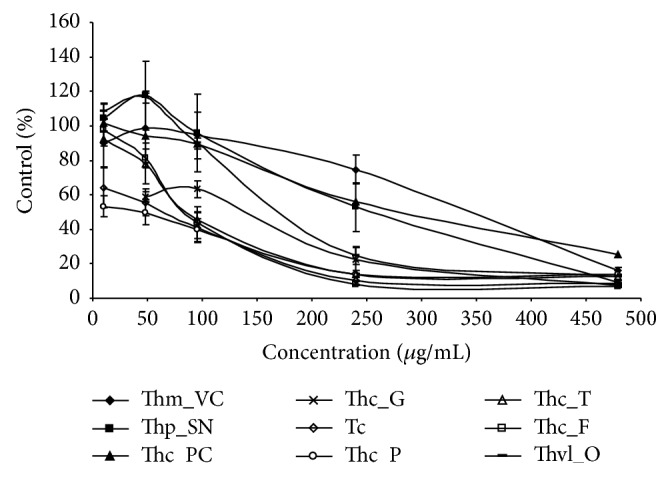
Antiproliferative activity of the essential oils on THP-1 cell line with 96 h exposure. The mean absorbance values for the negative control (DMSO treated cells) were standardized as 100% absorbance (i.e., no growth inhibition) and results were displayed as absorbance (% of control)* versus* essential oil concentration.

The MTT assay is a sensitive, simple, and reliable method for evaluating antiproliferative activity of plant-based products. The cytotoxic activities of the essential oils of* Thymbra* and* Thymus* species from Portugal were studied with the THP-1 leukemia cell line by treating these cells with increasing amounts of the essential oils for 24 and 96 h (Figures 2 and 3). In both cases, essential oils decreased viability of THP-1 cells in a dose-dependent manner.

After one day (24 h), a great difference was observed between the cytotoxicities of the EOs from* Th. mastichina* and* Th. caespititius* from Gerês and even more from that of Pico (Figure 2). These differences were detected even at low concentrations (<50 *μ*g/mL). At 10 *μ*g/mL, only 66% of THP-1 cells survived in the presence of the EO from* Th. caespititius* from Pico. At higher concentrations (>400 *μ*g/mL), EOs from* Th. mastichina*,* Th. pulegioides*,* Th. caespititius* from Praia do Cortiço, and* Th. villosus* showed the lowest cytotoxicity (Figure 2). 1,8-Cineole, geraniol, *α*-terpineol, and linalool were the main components of these EOs. Although thymol was present in low percentages in some samples (Thm_VC and Thp_SN), this was not enough for inhibiting the growth of THP-1 cells. Only EOs with higher thymol and carvacrol percentages were effective in preventing cell proliferation.

After four days (96 h), about 50% of THP-1 cells' survival was observed when exposed to 50 *μ*g/mL of* Th. caespititius* from Pico and* T. capitata* carvacrol rich EOs (Figure 3). At 100 *μ*g/mL, the survival was about 40%. The same survival percentage was observed for* Th. caespititius* from Terceira and Faial EOs with thymol and carvacrol as main components, respectively. At that concentration, the survival of cells was even about 90% in the presence of* Th. mastichina*,* Th. pulegioides*, and* Th. villosus* EOs. At 250 *μ*g/mL of EOs from* Th. caespititius* from Pico, Terceira, and Faial and* T. capitata*, only about 10% of THP-1 cells survived. With EOs from* Th. caespititius* from Praia do Cortiço,* Th. pulegioides*, and* Th. mastichina*, the survival percentages were still >50%, mainly that of* Th. mastichina* EO (>70%).

These results support the importance of carvacrol and thymol among EOs components, since when present at low percentages the EOs did not inhibit the growth of THP-1 cells. The antiproliferative activity of thymol and carvacrol as well as* Th. vulgaris* EO against THP-1 cells was also reported by Aazza et al. [17].* Origanum onites* carvacrol rich EO, between 62.5 and 125 *μ*g/mL, also presented toxicity against 5RP7 cancer cells (c-H-ras transformed rat embryonic fibroblasts) [20]. Also,* Satureja sahendica* thymol rich EO significantly reduced cell viability of the human colon adenocarcinoma (SW480), human breast adenocarcinoma (MCF7), choriocarcinoma (JET 3), and monkey kidney (Vero) cell lines [21].

## 4. Conclusions

In the Portuguese* Thymbra* and* Thymus* EOs studied, two main clusters were identified: one cluster grouping 8 samples with diverse percentages of carvacrol, *α*-terpineol, thymol,* p*-cymene, and *γ*-terpinene and the other cluster with only one EO in which linalool predominated and thymol and carvacrol were absent.

EOs with higher percentages of thymol and carvacrol showed the highest capacity for scavenging free radicals and preventing the growth of THP-1 cells.

## Figures and Tables

**Table 1:** Plant species scientific names, arranged according to alphabetic order, collection site, and corresponding code.

Plant species	Collection site	Code
*Thymbra capitata* (L.) Cav.	Gambelas, mainland Portugal	Tc
*Thymus caespititius* Brot.	Faial, Azores, Portugal	Thc_F
*Thymus caespititius* Brot.	Pico, Azores, Portugal	Thc_P
*Thymus caespititius* Brot.	Terceira, Azores, Portugal	Thc_T
*Thymus caespititius* Brot.	Gerês, mainland Portugal	Thc_G
*Thymus caespititius* Brot.	Praia do Cortiço, mainland Portugal	Thc_PC
*Thymus mastichina* (L.) L.	Vila Chã, mainland Portugal	Thm_VC
*Thymus pulegioides* L.	Serra da Nogueira, mainland Portugal	Thp_SN
*Thymus villosus* subsp. *lusitanicus* (Boiss.) Cout.	Óbidos, mainland Portugal	Thvl_O

**Table 2:** Percentage composition of the essential oils isolated from the aerial parts of *Thymbra* (Tc) and *Thymus* (Th) Portuguese species evaluated. Samples arranged according to the degree of correlation obtained after agglomerative cluster analysis based on the essential oils' chemical composition. For abbreviations and cluster analysis see Table 1 and Figure 1, respectively.

Components	RI	I								II
		Ia			Ib	Ic		Id		
		Tc	Thc_F	Thc_P	Thp_SN	Thc_T	Thm_VC	Thc_G	Thc_PC	Thvl_O
Tricyclene	921				0.1	t	t	0.1	0.1	0.1
*α*-Thujene	924	1.0	1.7	2.7	1.1	1.9	0.7	2.3	1.6	0.1
*α*-Pinene	930	1.0	0.7	0.9	1.4	0.6	2.4	1.8	0.9	0.7
Camphene	938	0.1	0.1	0.1	2.2	0.1	0.7	2.1	0.9	0.5
Thuja-2,4(10)-diene	940		0.1	0.1		0.1	t	t		
Sabinene	958		0.2	0.2	0.1	0.2	1.3	3.6	0.8	0.2
1-Octen-3-ol	961	0.4	t	t	0.4	t		0.4	0.3	t
3-Octanone	961				1.9					
*β*-Pinene	963	0.1	0.2	0.3		0.2	3.1	0.3	0.6	0.5
Dehydro-1,8-cineole	973		t			t	0.1	0.1	0.1	0.2
2-Pentyl furan	973		t			t		0.1	0.1	
3-Octanol	974	t			1.0	t	t	1.1	0.5	0.4
*β*-Myrcene	975	2.5			1.0	t	1.5	1.1	1.5	1.1
*α*-Phellandrene	995	0.3	0.1	0.2	0.2	0.2	0.3	0.3	0.1	0.2
*δ*-3-Carene	1000	0.1	0.1	0.1	t	0.1	t	0.1	0.1	
*α*-Terpinene	1002	1.8	0.6	0.8	1.0	1.3	0.8	1.6	0.6	0.1
*p*-Cymene	1003	8.8	5.8	7.3	6.4	13.5	9.7	12.2	10.4	3.0
1,8-Cineole	1005				1.5		47.4			6.3
*β*-Phellandrene	1005	0.4	0.2	0.3		0.2		0.7	0.2	
Limonene	1009	0.3	0.5	0.5	0.3	0.4	1.3	2.3	1.6	t
*cis*-*β*-Ocimene	1017				t	t	t	t	t	t
*trans*-*β*-Ocimene	1027	0.1			t	t	0.2	t		0.7
*γ*-Terpinene	1035	5.9	3.3	3.3	10.6	6.0	7.3	10.6	4.1	0.3
*trans*-Sabinene hydrate	1037	0.1	0.1		0.2	0.1	0.3	t	0.1	
*cis*-Linalool oxide	1045						t			0.9
Fenchone	1050						0.3			
*trans*-Linalool oxide	1059									0.8
*p*-Cymenene	1059						t			
2,5-Dimethyl styrene	1059				t	t		0.6	t	
Terpinolene	1064	0.2	0.2	0.2	0.1	0.1	0.1	0.4	0.4	
*cis*-Sabinene hydrate	1066	0.1			t	t	t	t	t	0.2
Linalool	1074	1.1			0.5	t	1.6	t	0.1	65.5
Oct-1-en-3-yl acetate	1086		0.2			0.6			0.2	
*trans*-*p*-2-Menthen-1-ol	1099				t	t	t	0.1	0.1	
Camphor	1102				2.3		0.3			0.5
*trans*-Pinocarveol	1106				t		0.1			
*cis*-*p*-2-Menthen-1-ol	1110					t		t		
*cis*-Verbenol	1110				t	t	t	t		
Pinocarvone	1121						t			
Nerol oxide	1127				t					
*p*-Mentha-1,5-dien-8-ol^*∗*^	1134							0.9		
*δ*-Terpineol	1134						0.7			0.4
Borneol	1134	0.1	0.1	t	1.0	0.1	0.7	0.9	1.2	0.4
Terpinen-4-ol	1148	0.8	0.8	1.0	0.4	0.7	0.7	1.9	1.0	0.5
*p*-Cymen-8-ol	1148					t		0.3		
Myrtenal	1153						t			
*cis*-Dihydrocarvone	1159				t					
*α*-Terpineol	1159	0.1	9.5	4.4	0.1	2.5	1.7	35.8	43.5	6.9
Methyl chavicol	1163						t			
Myrtenol	1168						t			
*trans*-Carveol	1189					0.1	t	t		
Bornyl formate	1199		0.1			t	t	t	0.1	
Nerol	1206				0.8					
Citronellol	1207	0.1								
Carvone	1210	0.1				t				
Thymol methyl ether	1210				t					
Neral	1210				0.2					
Carvacrol methyl ether	1224		1.1	0.1	0.3	t		0.2	0.4	
Geraniol	1236	t			32.8					
Geranial	1240	0.1			0.3					
*trans*-Anethole	1254						t			
Thymol formate	1262	0.1					t			
Bornyl acetate	1265			t	t	t	t	1.2	0.6	
Thymol	1275	0.4	0.1	10.3	12.0	42.2	13.7	t	0.3	
Carvacrol	1286	71.4	50.5	45.5	12.4	2.8	0.7	0.2	0.1	
Thymyl acetate	1330			2.4	t	15.2				
*δ*-Elemene	1332		0.5	0.5		0.4				
*α*-Terpenyl acetate	1334						0.4			
Carvacryl acetate	1348	0.1	5.9	12.3		0.7				
Geranyl acetate	1370				4.3					0.2
*α*-Copaene	1375					t		t	0.1	
*β*-Bourbonene	1379				0.3	t	t	0.2	0.2	0.6
*β*-Elemene	1388					0.1	t	0.1	0.3	t
*α*-Gurjunene	1400					t		t		
*β*-Caryophyllene	1414	1.6	0.1	0.1	1.2	t	0.6	1.0	1.2	0.7
*β*-Copaene	1426				t	t		t	0.1	
*trans*-*α*-Bergamotene	1434	t								
*cis*-Muurola-3,5-diene^*∗*^	1445					0.1				
*α*-Humulene	1447	0.1		t	t	t		0.1	0.1	0.1
*allo*-Aromadendrene	1456		0.5	0.4		0.4	t	0.3	0.6	t
*γ*-Muurolene	1469		0.1			t	0.1	t	0.1	
Germacrene-D	1474			t	0.7	0.1		0.7	0.6	0.1
*γ*-Humulene	1477			t				t		
Eremophilene^*∗*^	1480					0.1			0.1	0.2
Bicyclogermacrene	1487						0.1			0.7
Viridiflorene	1487						t			0.5
*trans*-Dihydroagarofuran	1489		2.7	0.7		0.8		0.4	0.5	
*α*-Muurolene	1494		0.2	0.2		0.4		0.2	0.4	
*β*-Bisabolene	1500	0.2			0.6					
*γ*-Cadinene	1500		1.9	0.9		1.2	t	1.3	2.9	
*trans*-Calamenene	1505		0.4	0.1		0.3		0.2	0.2	
*δ*-Cadinene	1505		0.4	0.4		0.2	t	0.3	0.1	
Kessane^*∗*^	1517		1.3	0.2		0.3		0.3	1.6	
*α*-Calacorene	1525		t	t		t		t		
*α*-Cadinene	1529		0.1	0.1		t		0.1	0.9	
Elemol	1530		0.1	t		t	0.1	0.6	0.1	0.2
*trans*-*α*-Bisabolene	1536	0.2								
Geranyl butyrate	1544				0.1		t			
Spathulenol	1551		t	t		t	0.1	0.1	0.3	0.2
*β*-Caryophyllene oxide	1561	0.1	t	t		t	t	0.1		0.3
Globulol	1566			t		t		0.8		
Geraniol 2-methyl butyrate	1586				t					
10-epi-*γ*-Eudesmol	1593						t			
epi-Cubenol	1600		1.6	0.5		0.6		0.4	0.6	
*γ*-Eudesmol	1609		0.1	0.1		t		0.5	0.7	0.1
*τ*-Cadinol	1616		3.3	1.2		2.5	t	4.8	6.7	0.8
*α*-Muurolol	1618		0.1	0.1		t		0.3	0.6	
*β*-Eudesmol	1620		0.1	0.1		t	t	0.3	0.6	0.1
Intermedeol	1626						t			3.4
*α*-Eudesmol	1634		0.4	0.4		1.3		1.3	2.4	
*α*-Bisabolol	1656					t				
Rosadiene^*∗*^	1993	0.1								
Abietatriene	2027	t								

*% identification *		99.9	96.1	99.0	99.8	98.7	99.1	97.7	94.6	98.7

*Grouped components *										
Monoterpene hydrocarbons		22.6	13.8	17.0	24.5	24.9	29.4	39.5	23.9	7.5
Oxygen-containing monoterpenes		74.6	68.2	76.0	69.2	64.4	68.7	41.6	47.6	82.8
Sesquiterpene hydrocarbons		2.1	4.2	2.7	2.8	3.3	0.8	4.5	7.9	2.9
Oxygen-containing sesquiterpenes		0.1	9.7	3.3		5.5	0.2	9.9	14.1	5.1
Diterpenes		0.1								
Phenylpropanoids							t			
Others		0.4	0.2	t	3.3	0.6	t	2.2	1.1	0.4

All components were identified based on a lab-made library created with reference essential oils, laboratory-synthesized components, laboratory isolated compounds, and commercial available standards. RI: in-lab obtained retention index relative to C_9_–C_21_ *n*-alkanes on the DB-1 column; t: traces (<0.05%). ^*∗*^Tentative identification based only on mass spectra.

**Table 3:** Antioxidant activity of essential oils evaluated by the TEAC and ORAC methods.

Plant species	Code	TEAC (*μ*mol TE/g essential oil)	ORAC (*μ*mol TE/g essential oil)
*Thymbra capitata *	Tc	25.2 ± 1.3^ab^	183.6 ± 9.6^a^
*Thymus caespititius *	Thc_F	25.8 ± 1.3^ab^	182.8 ± 9.6^a^
*Thymus caespititius *	Thc_P	23.0 ± 1.3^ab^	170.3 ± 9.6^abc^
*Thymus caespititius *	Thc_T	27.3 ± 1.3^a^	190.6 ± 9.6^a^
*Thymus caespititius *	Thc_G	10.8 ± 1.3^c^	144.5 ± 9.6^cd^
*Thymus caespititius *	Thc_PC	8.1 ± 1.3^c^	127.1 ± 9.6^d^
*Thymus mastichina *	Thm_VC	21.2 ± 1.3^b^	178.4 ± 9.6^ab^
*Thymus pulegioides *	Thp_SN	22.8 ± 1.3^ab^	179.4 ± 9.6^ab^
*Thymus villosus* subsp. *lusitanicus *	Thvl_O	3.7 ± 1.3^d^	148.4 ± 9.6^bcd^

Values in the same column followed by the same letter are not significant by Tukey's multiple range test (*p* < 0.05).
